# A disseminated variant of pancreatic serous cystadenoma causing obstructive jaundice, a very rare entity: a case report and review of the literature

**DOI:** 10.1186/1756-0500-7-749

**Published:** 2014-10-22

**Authors:** Bilal O Al-Jiffry, Fares Rayzah, Samah H Khayat

**Affiliations:** Department of Surgery, Taif University, PO Box 888, Taif, 21947 Kingdom of Saudi Arabia; Department of Surgery, AlHada Military Hospital, Taif, Saudi Arabia

**Keywords:** Pancreatic cysts, Disseminated variant, Serous cystadenoma, Obstructive jaundice

## Abstract

**Background:**

Microcystic adenoma or serous cystadenoma (SCA) is an uncommon tumor type, accounting for only 1–2% of pancreatic exocrine neoplasms. Usually unifocal, SCAs present as single, large, well-demarcated, multiloculated, cystic tumors, 1–25 cm in size.

**Case presentation:**

A 73-year-old man initially presented with epigastric abdominal pain and was diagnosed with SCA involving the whole pancreas. Eleven months later, he presented with obstructive jaundice, and total pancreatectomy was performed. The removed tissue allowed histological verification of pancreatic SCA. Histopathological examination showed both microcysts and macrocysts, lined by cuboidal epithelium, with optically clear cytoplasm and the absence of detectable mitosis or necrosis.

**Conclusions:**

Thus, although relatively rare, pancreatic SCA is one of the differential diagnoses of epigastric abdominal pain; we recommend early surgical intervention for symptomatic pancreatic SCA.

## Background

Microcystic adenoma or serous cystadenoma (SCA) of the pancreas is an uncommon, benign tumor that accounts for 1–2% of pancreatic exocrine neoplasms and 25% of cystic neoplasms [1]. Their prevalence is increasing, primarily due to imaging technique improvements. These tumors are usually unifocal, mostly arising in the body and tail of the pancreas and are often incidentally detected. They present as single, large, well demarcated, and multiloculated cystic tumors, 1–25 cm in size. They are composed of cysts lined by epithelial cells that produce serous fluid and show ultrastructural evidence of centroacinar differentiation [2].

The World Health Organization (WHO) has subclassified these tumors into two groups, serous microcystic adenomas and serous oligocystic adenomas. The serous microcystic adenoma is a well-demarcated tumor composed of numerous small cysts arranged around a central stellate scar. The central stellate scar is a dense fibrous core within the tumor, which may be calcified and from which fibrous trabeculae radiate to the periphery. The serous oligocystic adenoma is an often ill-demarcated tumor composed of a few cysts with diameters of 1–2 cm. Serous cystic tumors showing larger cysts have been referred to as serous macrocystic adenomas [3]. A third type, the disseminated variant (DV), has been recognized because of its rare involvement of the entire pancreas. In the DV, both of the previously described types are spread throughout the pancreas. Although DVs are rare, they are more commonly reported in association with Von Hippel-Lindau (VHL) disease [4]. VHL disease, which is an autosomal dominant condition having variable penetrance, is characterized by central nervous system (CNS) and retinal hemangioblastomas, visceral cysts, pheochromocytomas, and renal cell carcinoma; pancreatic lesions are seen in approximately 56% of VHL patients [4]. Criteria for the diagnosis of VHL include the presence of a CNS hemangioblastoma with at least one other VHL-type lesion in an individual or one VHL-type lesion in a family member if another family member has a CNS hemangioblastoma [4].

Here, we present a rare case of a pancreatic SCA involving the whole pancreas **(**DV) that caused obstructive jaundice, with no association with VHL.

## Case presentation

**Figure 1:**
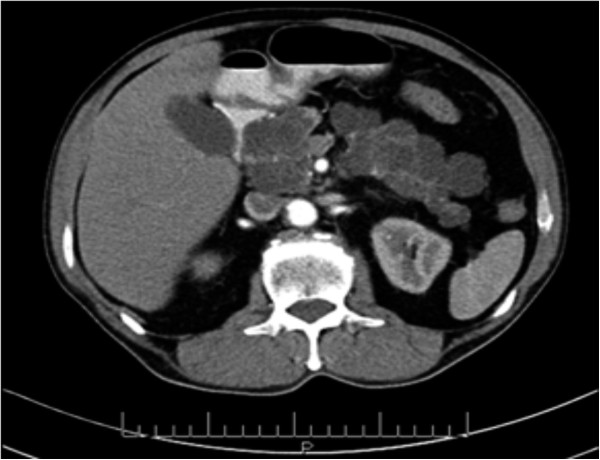
**Contrast enhanced computed tomography showing multiple, variably sized cystic lesions with multiple calcific foci involving the whole pancreas.**

**Figure 2:**
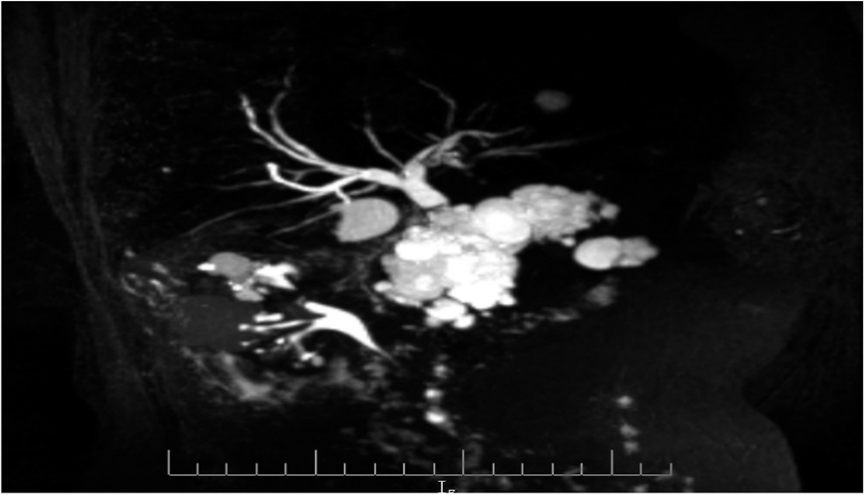
**Magnetic resonance cholangiopancreatography showing numerous pancreatic cysts involving the entire pancreas, obscuring the pancreatic duct.**

A 73-year-old man, with a history of diabetes mellitus and hypertension, was referred to our clinic in November 2010 with a 3-year history of epigastric pain that was increasing in severity. The pain was non-colicky and was not associated with jaundice or weight loss. The patient did not have any significant personal or family history with no previous admissions and had not been previously admitted for investigation of these symptoms. General and systemic examinations were normal and his serum biochemistry, including liver function tests, amylase and lipase tests were within normal limits, except for his carbohydrate antigen (CA 19–9), which was 60 U/mL (normal range 0–10 U/ml). Abdominal computed tomography (CT) (Figure 1) showed multiple, variably sized cystic lesions with multiple calcific foci involving the whole pancreas (Figure 1). Magnetic resonance imaging (MRI) and magnetic resonance cholangiopancreatography (MRCP) showed numerous pancreatic cysts involving the entire pancreas, obscuring the pancreatic duct (Figure 2). A diagnosis of diffuse SCA was made and he was offered surgical intervention; he declined and sought a second opinion.

**Figure 3:**
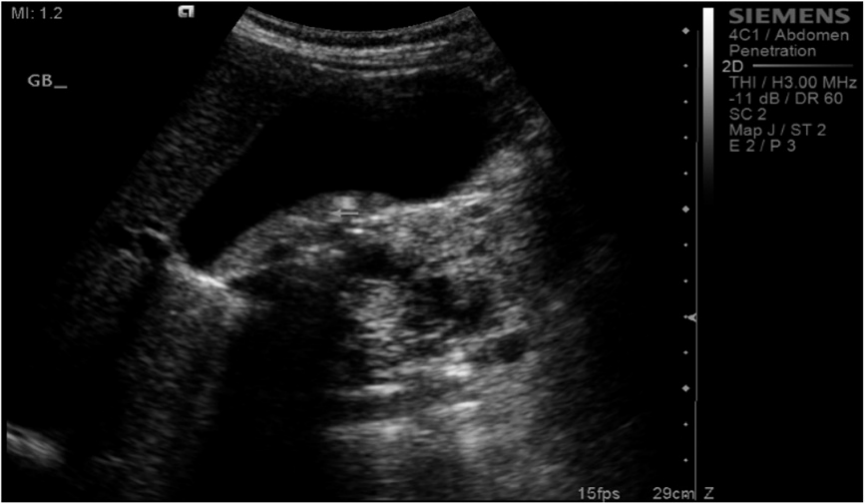
**Abdominal ultrasonography showing multiple pancreatic cysts and a dilated biliary system with common bile duct stones.**

Approximately 1 year later, he presented with obstructive jaundice and abnormal serum biochemistry results: alkaline phosphatase, 218 U/L (normal range, 30–120 U/L); total bilirubin, 82.2 μmol/L (normal range, 0–20 μmol/L); direct bilirubin, 51.2 μmol/L (normal range 0–15 μmol/L) ; aspartate aminotransferase (AST), 177 U/L (normal range, 0–45 U/L); alanine aminotransferase (ALT), 260 U/L (normal range, 0–45 U/L); serum amylase, 37 U/L (normal range, 30–110 U/L); serum lipase, 11 U/L (normal range, 5–208 U/L), and CA 19–9, 60 U/mL (normal range, 0–37 U/mL). Abdominal ultrasonography showed multiple pancreatic cysts and a dilated biliary system with common bile duct (CBD) stones (Figure 3). Endoscopic retrograde cholangiopancreatography (ERCP) also showed severe distal CBD narrowing, and the stones were extracted. Another abdominal CT was done because of the elapsed time of one year with no follow up. It showed the absence of any malignant changes and an increase in the size of some cysts. VHL disease was excluded, based on a normal neurological examination, normal visual acuity, and the absence of other organ involvement, including a normal CT brain. Again, the patient was offered surgical treatment, but he asked for additional time to consider this option and was discharged without jaundice and in a normal condition.

**Figure 4:**
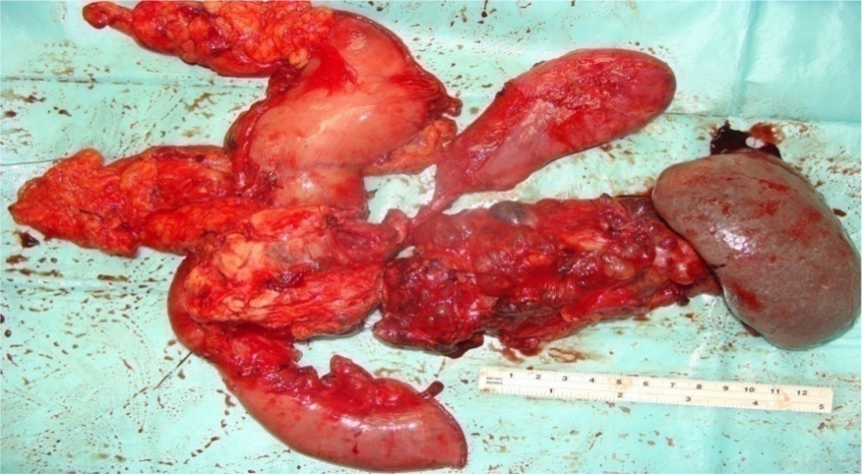
**Total pancreatectomy specimen showing diffuse replacement of the pancreas with multiple cysts.**

Two months later, he presented again with obstructive jaundice. MRI and MRCP were repeated to evaluate the CBD and demonstrated dilatation of the intrahepatic and extrahepatic biliary systems, but no stones. The patient agreed to surgery and underwent total pancreatectomy. Intraoperatively, the entire pancreas (including the uncinate process) was distorted and replaced by variably sized cysts, up to 3 cm (Figure 4). Remarkably, the operation was uneventful without evidence of other organ or lymph node involvement. The patient’s postoperative course was uneventful and he was discharged 10 days later. The patient is currently taking pancreatic enzyme replacements, and is alive and well 20 months after surgery.

**Figure 5:**
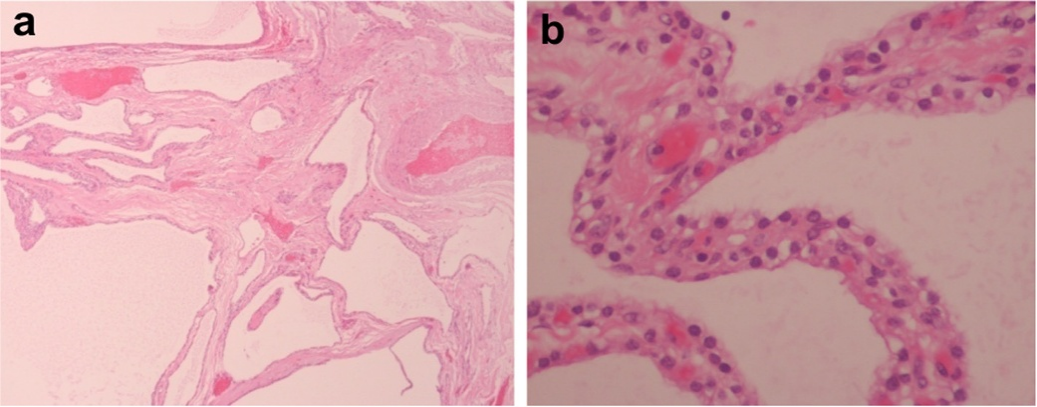
**Microscopic examination of the pancreatic specimen a) showing the presence of microcysts and macrocysts, b) The wall lined by cuboidal epithelium and possessing optically clear cytoplasm without any evidence of mitosis or necrosis.**

A microscopic examination (Figure 5) of the pancreatic specimen showed microcysts and macrocysts lined by cuboidal epithelium, with optically clear cytoplasm and the absence of evident mitosis or necrosis.

## Discussion

SCAs are benign cystic tumors composed of cuboidal epithelium and producing serous fluid [3]. Although rare cases of serous cyst adenocarcinoma have been reported [5], the WHO reported that the prevalence of malignant pancreatic SCAs is 1–3% [5]. This malignant variant is defined by the presence of metastases in extrapancreatic organs or tissues [5]. In addition, the presence of invasion to the adjacent organs is considered a characteristic of malignancy [6]. SCAs predominate in females in their sixth decade of life [7] and are asymptomatic in 33% of cases [7]. Symptoms are frequently non-specific, with abdominal pain being the most common, occurring in 50–60% of all cases [7]. The tumors rarely cause obstructive jaundice [6, 8–12] and are usually unifocal, situated in the body and tail of the pancreas, and are multiloculated and cystic in appearance. The present case can be classified as one of a multicentric serous tumor involving the entire pancreas with almost complete obliteration of pancreatic acini and ducts.

DV pancreatic lesions have been reported in up to 56% of patients with VHL disease [4], which is an autosomal dominant genetic disorder. The VHL tumor suppressor gene, linked to chromosome 3p 2 s, has been identified. When both copies of this gene are inactivated, there is unchecked cell growth resulting in tumors and cysts in the pancreas, kidney, and epididymis. Pancreatic manifestations of VHL are frequently asymptomatic and consist of SCA or multiple serous cysts, sometimes replacing the entire pancreas [4]. Reports of SCA, without a family history of VHL, have also been cited, in part probably due to sporadic inactivation of the gene [4]. An extensive review of the literature revealed only 16 reported cases of SCA associated with obstructive jaundice (including 1 DV case) [7–12]; 9 were in women and 7 in men, with a mean age of 59.5 years. However, only 9 cases of DV involving the entire pancreas have been reported in literature published in English language journals (Table 1). Thus, to the best of our knowledge, this is only the second case of DV pancreatic SCA without VHL causing obstructive jaundice. The differential diagnosis of SCA from pseudocysts and other cystic neoplasms is very important because, in contrast to other cystic neoplasms, SCAs may not require surgery. Serous microcystic adenomas can be misdiagnosed as solid lesions if small loculi with septa cannot be visualized [13]. Serous oligocystic adenomas can be misdiagnosed as either mucinous cystic tumors or pseudocysts because of their oligocystic or unilocular nature [13]. However, most patients with pseudocysts have a history of pancreatitis and they are often older individuals. Hypervascularity is another feature distinguishing SCA from pseudocysts [14]. Endoscopic retrograde pancreatography shows the communication of the pseudocyst with the pancreatic duct in 70% of the cases, in contrast to SCAs, which lack such a communication [13]. On CT scans, mucinous cystic tumors have a few, large loculi with thin septa. In contrast, SCAs have intratumoral calcification; mucinous tumors and pseudocysts may have peripheral calcification [14].

**Table 1:** **Patients with diffuse pancreatic serous cystadenoma (SCA)**

No	Group (first author)	Age/sex	Site/number	Malignant manifestations	Associated tumors	Associated jaundice	Procedure performed	Follow up
1	Compagno, 1978 [7]	NA	3 cases	No	No	Yes		
2			SCA-diffuse			(One case)		
3							NA	NA
4	Shorten, 1986 [15]	60/F	SCA–diffuse	No	No	No	Core needle biopsy only	A & W, 2 yr
5	Kim, 1990 [16]	32/F	SCA–diffuse	No	No	No	Subtotal pancreatectomy	A & W 3 months
6	Kim, 1997 [17]	67/F	SCA–diffuse	No	Pancreatic endocrine tumor	No	Whipple’s procedure	NA
7	Yasuhara, 2002 [18]	51/M	SCA–diffuse	No	No	No	Total pancreatectomy	A & W 3 months
8	Tampi, 2006 [19]	36/F	SCA–diffuse	No	No	No	Subtotal pancreatectomy	NA
9	Agarwal, 2009 [20]	35/F	SCA–diffuse	No	Neuroendocrine carcinoma	No	Total pancreatectomy	NA

NA, not available; A & W, alive and well.

Differentiation of SCAs from mucinous cystadenomas and pseudocysts is usually based on ultrasonography or CT findings. When the distinction is less clear, fine-needle aspiration of the cystic lesions for cytology and biochemical analysis of the fluid may help in establishing the diagnosis. On cytological analysis, periodic acid-Schiff stains of SCAs show abundant cytoplasmic glycogen, and stains for mucin are negative [21]. Cells are cuboidal to polygonal in shape, without evident mitoses; cytological analysis is diagnostic in about 20% of cases [22]. Biochemical analysis of cyst fluid may include tests for amylase and some tumor markers, such as carcinoembryonic antigen, NB/70K, CA 72–4, CA 125, CA 15–3 tissue polypeptide antigen, and pS2 protein [23, 24]. The amylase content of the fluid in SCAs is usually lower than the levels in pseudocysts and mucinous cystic tumors, and SCAs have lower levels of tumor markers compared with mucinous cystic tumors.

Most authors advise that asymptomatic patients be closely monitored, so long as potentially malignant cystic tumors, mainly mucinous cystadenoma or intraductal papillary mucinous tumor, can be definitively excluded. Surgical intervention is indicated for patients with symptomatic lesions and those with an uncertain diagnosis [25, 26]. Size of the cyst at presentation is still debatable since some reports indicate resection for cysts >4 cm and others do not [27].

Recently published guidelines for cystic pancreatic management suggest that resection should be considered for all symptomatic cases of SCA, for patients with cysts >4 cm, in case of presence of mural nodules, dilatation of main pancreatic duct >6 mm, increased serum levels of carbohydrate antigen (CA 19–9), or in case of rapid increase in size [28, 29].

## Conclusions

In conclusion, SCAs of the pancreas are increasing in frequency of diagnosis because of advances in imaging techniques. The differentiation of SCAs from other cystic tumors, as well as from non-neoplastic cysts, is very important because of the great differences in the management of these different entities. This case highlights an extremely rare case of SCA involving the whole pancreas (DV) causing obstructive jaundice, without an association with VHL disease.

## Consent

Written informed consent was obtained from the patient for publication of this Case report and any accompanying images. A copy of the written consent is available for review by the Editor of this journal.
